# Thanking cell and bioscience reviewers

**DOI:** 10.1186/s13578-016-0117-3

**Published:** 2016-09-07

**Authors:** Yun-Bo Shi

**Affiliations:** Section on Molecular Morphogenesis, NICHD, National Institutes of Health, Bethesda, MD 20892 USA

## Contributing reviewers

The editors of cell and bioscience would like to thank all the reviewers who contributed to the journal in 2015.

**Kannan Alpadi**

USA

**Ingrun Alseth**

Norway

**Francesco Bernardi**

Italy

**Paul Brehm**

USA

**Daniel Buchholz**

USA

**Qiliang Cai**

China

**Kaixiang Cao**

USA

**Bryce Chackerian**

USA

**Chih-Chiang Chan**

Taiwan

**Chuang-Rung Chang**

Taiwan

**Rui Chang**

USA

**Wei-June Chen**

Taiwan

**Ruey-Hwa Chen**

Taiwan

**Guoxun Chen**

USA

**Didi Chen**

USA

**Pei-Yu Chen**

USA

**Wanjun Chen**

USA

**Xiaolei Chen**

USA

**Steven Cheng**

China

**Linzhao Cheng**

USA

**Christine Cheung**

Singapore

**Ya-Hui Chi**

Taiwan

**Tian Chi**

USA

**Justin Jang Hann Chu**

Singapore

**Yingzi Cong**

USA

**Javier Corral De La Calle**

Spain

**Toos Daemen**

Netherlands

**David Davido**

USA

**Hua Deng**

USA

**Changwang Deng**

USA

**Qiurong Ding**

China

**Sheng Ding**

USA

**Junjun Ding**

USA

**Jihui Du**

China

**Dan Du**

USA

**Zhanwen Du**

USA

**Elia Duh**

USA

**Wenwen Fang**

USA

**Xin-Hua Feng**

USA

**Archa Fox**

Australia

**Clara Franzini-Armstrong**

USA

**Xianghui Fu**

China

**Chuanhai Fu**

Hong Kong

**Qiang Gan**

USA

**Guoquan Gao**

China

**Ouyang Gaoliang**

China

**Xinquan Ge**

USA

**Deming Gou**

China

**Alexei Gratchev**

Russia

**Ai-Di Gu**

USA

**Dongyin Guan**

USA

**Yongjun Guo**

China

**Li-Tao Guo**

USA

**Xiaoli Guo**

USA

**Brett Hambly**

Australia

**Zhipeng Han**

China

**Weiping Han**

Singapore

**Zhe Han**

USA

**Takashi Hasebe**

Japan

**Johnny He**

USA

**Yunlong He**

USA

**Jian Hong**

China

**Kuo-Sheng Hsu**

USA

**Jim Hu**

Canada

**Jian Huang**

China

**Jing Huang**

USA

**Yi-Wen Huang**

USA

**Kam Man Hui**

Singapore

**Lihong Huo**

USA

**Yong Ji**

China

**Li–Li Jiang**

China

**Xiuyun Jiang**

USA

**Dong-Yan Jin**

Hong Kong

**Qing Jing**

China

**Hung-Ying Kao**

USA

**Joel Karliner**

USA

**Liu Keli**

China

**Juliann Kiang**

USA

**Tatsuo Kido**

USA

**Kin-Hang Kok**

Hong Kong

**Ah-Ng Tony Kong**

USA

**Fouad Lafdil**

France

**Amale Laouar**

USA

**Peng Lee**

USA

**Qiao Li**

Canada

**Lc Li**

China

**Qing Li**

China

**Wenhua Li**

China

**Wenxin Li**

China

**Jun Li**

USA

**Shengwen Calvin Li**

USA

**Chengyu Liang**

USA

**Ming-Fong Lin**

USA

**Shengda Lin**

USA

**Ying-Bin Liu**

China

**Jinsong Liu**

USA

**Ping Liu**

USA

**Xiang Liu**

USA

**Zheng-Gang Liu**

USA

**Lei Lu**

Singapore

**Vivian Lui**

Hong Kong

**Shiwen Luo**

China

**Hua Luo**

USA

**Kai Mao**

USA

**Craig Mclachlan**

Australia

**Zhipeng Meng**

USA

**Sujit Nair**

India

**Wen Pan**

USA

**Dai-Wen Pang**

China

**Zhiping Pang**

USA

**Suman Paul**

USA

**Thomas Pressley**

USA

**Yitao Qi**

USA

**Xuebin Qin**

USA

**Jianming Qiu**

USA

**Yun Qiu**

USA

**Fangfang Qu**

USA

**Harish Ramanathan**

USA

**Amaswamy Ramchandran**

USA

**Liangyou Rui**

USA

**Amrik Sahota**

USA

**Jagan Sastry**

USA

**Inge Schmitz**

Germany

**Nilkantha Sen**

USA

**Ashwani Sharma**

USA

**Zhiyuan Shen**

USA

**Yufang Shi**

China

**Yuji Shi**

USA

**Yunbo Shi**

USA

**Peiqing Shi**

USA

**Hsiu-Ming Shih**

Taiwan

**Liu Shih-Jen**

Taiwan

**Xintao Shuai**

China

**Sanjeev Shukla**

USA

**Jay Singh**

USA

**Rohit Anthony Sinha**

Singapore

**Qingxuan Song**

USA

**Wenqiang Song**

USA

**Shao-Cong Sun**

USA

**Xiaojun Tan**

USA

**Yuliang Tan**

USA

**Bl Tang**

Singapore

**Dean Tang**

USA

**Fei Tang**

USA

**Sai-Wen Tang**

USA

**Yanmei Tao**

China

**Akihiro Tomita**

Japan

**Joshua Vieth**

USA

**Yisong Wan**

USA

**Shouli Wang**

China

**Tuanlao Wang**

China

**Ying Wang**

China

**Yuan Wang**

China

**Limin Wang**

USA

**Shaoying Wang**

USA

**Tao Wang**

USA

**Rongfu Wang**

USA

**Yimeng Wang**

USA

**Zhou Wang**

USA

**Lixin Wei**

China

**Qing Wei**

USA

**Luan Wen**

USA

**Jiemin Wong**

China

**Yuntao Wu**

USA

**Gutian Xiao**

USA

**Ting Xie**

USA

**Jianming Xu**

USA

**Wancai Yang**

USA

**Yoshio Yaoita**

Japan

**Peiying Ye**

USA

**Paul Yen**

Singapore

**Hao Ying**

China

**Zongbing You**

USA

**Siwang Yu**

China

**Zhongsheng Yu**

USA

**Kit San Yuen**

Hong Kong

**Qi Zeng**

USA

**Ge Zhang**

China

**Xiao-Dong Zhang**

China

**Zhenfeng Zhang**

China

**Li Zhang**

USA

**Lu Zhang**

USA

**Quanguang Zhang**

USA

**Mingliang Zhang**

USA

**Yanling Zhang**

USA

**Ying Zhang**

USA

**Hui Zhao**

Hong Kong

**Keji Zhao**

USA

**Xuan Zhao**

USA

**Zhen Zhao**

USA

**Richard Y. Zhao**

USA

**Chao Zheng**

USA

**Xingnan Zheng**

USA

**Zhi-Ming Zheng**

USA

**Rongjia Zhou**

China

**Beiyan Zhou**

USA

**Qiang Zhou**

USA

